# Adaptive Beam Splitting-Based Broadband Hybrid Precoding for Terahertz Massive MIMO

**DOI:** 10.3390/s23041968

**Published:** 2023-02-09

**Authors:** Lei Xu, Yu Liu, Jing Chang, Hongyu Fang, Xiaohui Li

**Affiliations:** School of Integrated Circuits, Anhui University, Hefei 230601, China

**Keywords:** terahertz massive MIMO, broadband hybrid precoding, beam splitting, beam tracking, beam aggregation

## Abstract

Terahertz massive MIMO systems can be used in the local area network (LAN) scene of maritime communication and has great application prospects. To solve the problems of excessive beam training overhead in beam tracking and beam splitting in beam aggregation, a broadband hybrid precoding (HP) is proposed. First, an additional delayer is introduced between each phase shifter and the corresponding antenna in the classical sub-connected HP structure. Then, by precisely designing the time delay of the delayer and the phase shift of the phase shifter, broadband beams with flexible and controllable coverage can be generated. Finally, the simulation results verify that the proposed HP can achieve fast-tracking and high-energy-efficient communication for multiple mobile users.

## 1. Introduction

With the fast development of the blue economy and the construction of smart oceans, maritime communications have attracted ever-increasing research attention [1,2,3]. Compared to the typical bandwidth of several GHz in the millimeter-wave (mmWave) band (30–100 GHz) for 5G, the terahertz (THz) band (0.1–10 THz) for 6G is capable of providing at least 10 GHz bandwidth or even much larger. Therefore, THz communication can be used in the LAN scene of maritime communication and has great application prospects. Since the attenuation of THz signals is greater than that of mmWave signals, it usually employs larger-scale MIMO antenna arrays than mmWave communication to compensate for signal attenuation. Precoding is a channel adaptive technique, which preprocesses the transmitted signal based on the channel information at the transmitter. Usually, THz precoding is separated by analog beamforming and digital precoding due to the application of a hybrid analog-digital precoding structure. However, the widely considered HP structure in massive MIMO cannot handle the beam splitting problem caused by the wideband and large number of antennas in THz massive MIMO systems [4].

For beam tracking, the beams generated by larger massive MIMO antenna arrays are narrower, a small position change may cause the beam to be unable to align with the user, making it difficult to achieve continuous coverage for mobile users. A scanning beam tracking technology widely used in 5G mmWave systems can be extended to THz massive MIMO systems [5]. However, it can only track a certain physical direction angle in each scan, resulting in excessive beam training overhead. Furthermore, it is difficult for the beam to accurately capture the trajectory of mobile users in a short period of time, resulting in the system performance being significantly degraded. A class of delay-phase precoding is proposed based on the classical sub-connected structure [6] and the classical fully-connected structure [7] of hybrid precoding, respectively. By introducing a certain number of delayers between the radio-frequency (RF) chain and the classical phase-shifter network, they can aggregate the naturally splitting beams into a certain physical direction range, and only need a few time slots to complete the traversal of the potential moving range centered with the user’s previous time slot position, thus greatly reducing the training overhead of beam tracking. However, it requires a priori condition of the user’s movement range and requires multiple time slots, which will lead to its inability to be directly applied to the real scene of the user’s rapid movement. For beam aggregation, the beam splitting question makes the traditional beam generated by the frequency-independent phase shifter of hybrid precoding achieve high array gain only near the center frequency. The array gain is seriously lost at most of the subcarrier frequencies, resulting in serious achievable sum-rate loss, and offset the performance gain from increased bandwidth. In order to compensate for the loss of array gain, a delay-phase precoding has been proposed to handle the beam splitting question by jointly controlling the delayers and phase shifters [8]. The aggregation of naturally split beams can be realized, so as to solve the beam splitting problem under a small broadband-frequency ratio. However, the large bandwidth is not considered in the design of time delay, so the effect is very limited under the large bandwidth-frequency ratio.

In summary, the introduction of frequency-dependent phase shift through the addition of a delayer provides the feasibility for the hybrid precoding structure to be effectively extended to THz communication. In particular, based on the basic characteristics of the beam splitting problem, adding a group of the delayers with the same number of base station antennas on each RF chain of the hybrid precoding with the fully-connected structure can perfectly solve the beam splitting problem, but it brings a sharp increase in energy consumption [9]. In addition, although the fully-connected structure of hybrid precoding can obtain full array gain, its structure is complex, and cannot be easy to make corresponding structural adjustments with the change of the user number. Therefore, the delay-phase precoding in [7,8] is more suitable for scenarios with a stable number of users.

In order to achieve a better tradeoff between achievable sum-rate and energy consumption, and improve the adaptability to the change of the number of users, a broadband hybrid precoding based on adaptive beam splitting is proposed. First, an additional delayer is added between each phase shifter and the corresponding antenna in the classical sub-connected HP structure [10], thereby the frequency-dependent phase shift is introduced. Then, in the beam tracking, the degree of beam splitting is increased by the delayer, and the beam of the RF chain is split into several sub-beams of subcarriers by the determination of analog precoding, so as to realize the simultaneous and global tracking for multiple users. Finally, in the beam aggregation, the degree of beam splitting is reduced by the delayer, and the naturally split sub-beams on each RF chain are aggregated in the corresponding user physical direction through the determination of analog precoding, so as to greatly improve the available sum-rate performance.

The main contributions of this paper can be summarized as follows: (1) In order to realize adaptive beam splitting, achieve a better tradeoff between achievable sum-rate and energy consumption, and improve the adaptability to the change of the number of users, the broadband HP framework based on adaptive beam splitting is proposed. (2) In the beam tracking, a low complexity beam tracking scheme is proposed based on the widely used frame structure of THz communication. The degree of beam splitting can be expanded by the delayers. Specifically, by jointly controlling time delay and analog precoding parameters, the beam of each RF chain is split into *M* sub-beams corresponding to *M* subcarriers, respectively, which can cover the whole tracking range aiming at detecting different user’s physical directions, so the beam tracking problem can be transformed into the low-complexity beam selection problem. (3) In the beam aggregation, based on the processing of beam tracking, the physical direction of each user is already known. Because the system achievable sum-rate performance is directly related to the array gain, the achievable rate maximization problem can be transformed into the array gain maximization problem. Through the design of time delay and analog precoding, the degree of beam splitting can be reduced and the *M* naturally split sub-beams on each RF link can be aggregated in the corresponding user physical direction. This can maximize the array gain at last.

The remainder of this paper is organized as follows. The broadband hybrid precoding framework based on adaptive beam splitting is presented in Section 2. The adaptive beam splitting methods in the beam splitting and the beam aggregation are investigated separately in Section 3 and Section 4. The system simulation results are discussed in Section 5. Finally, the conclusion is presented in Section 6.

## 2. System Model

**Figure 1:**
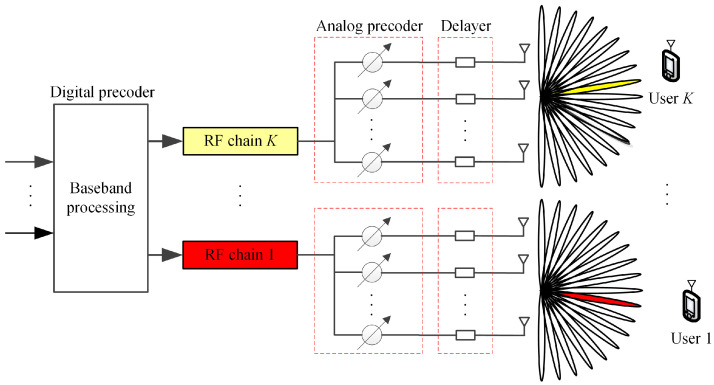
Broadband hybrid precoding framework based on adaptive beam splitting.

Broadband HP framework based on adaptive beam splitting is shown in Figure 1. We consider a THz massive MIMO communication system with the classical sub-connected HP structure. The base station employs an $N_{t}$-antenna uniform linear array and $N_{RF}$ RF chains, and each RF chain generates one beam to serve one single-antenna user. Assuming the number of RF chains is the same as the number of users ($N_{RF}=K$), and the orthogonal frequency division multiplexing (OFDM) with *M* subcarriers is adopted to realize broadband transmission. The received signal at the *m*-th (m∈[1,⋯,M]) subcarrier of the *k*-th (k∈[1,⋯,K]) user can be expressed as

(1) $$y_{k,m}=H_{m[k,:]}W_{m}Ad_{k,m}s_{k,m}+n_{k,m},$$

where $s_{k,m}$ is the transmitted signal at the *m*-th subcarrier of *k*-th user with power normalized as $|s_{k,m}{|}^{2}\;=\;1$. $d_{k,m}$ is the digital precoding vector of the *m*-th subcarrier and the *k*-th RF chain, which can be generated by (7). $n_{k,m}$ denotes the noise at *m*-th subcarrier of *k*-th user, obeying a complex Gaussian distribution with a mean value of 0 and a variance of $\sigma^{2}$. $H_{m}=\mathrm{diag}\left(h_{1,m},h_{2,m},\cdots ,h_{K,m}\right)\in C^{K\times N_{t}}$ presents the downlink channel matrix at the *m*-th subcarrier of all *K* users. For THz channels, the widely used ray-based wideband THz channel model is considered [11]. Since the path loss of non-line of sight (NLoS) is much larger than that of the line of sight (LoS) path, the influence of the NLoS path on the channel can be negligible [7]. Without loss of generality, each RF chain is connected to an *N*-antenna subarray, where *N* satisfies $N=N_{t}/K$ and is assumed to be an integer. Therefore, the downlink channel of the *k*-th user at *m*-th subcarrier $h_{k,m}\in C^{1\times N}$ can be simplified as

(2) $$h_{k,m}=\lambda_{k,m}e^{-j2\pi \tau_{k}f_{m}}f_{t}^{H}\left(\theta_{k,m}\right),$$

where $\lambda_{k,m}$ and $\tau_{k}$ represent the path gain and path delay of the LoS path between the base station and the *k*-th user, respectively. $f_{t}\left(\theta_{k,m}\right)\in C^{N\times 1}$ is the array response vector of the antenna subarray corresponding to an RF chain, which can be expressed as

(3) $$f_{t}\left(\theta_{k,m}\right)=\frac{1}{\sqrt{N}}{\left[1,e^{j\pi {\theta_{k}}_{,m}},\cdots ,e^{j\pi \left(N-1\right){\theta_{k}}_{,m}}\right]}^{T},$$

where the spatial direction of the beam is satisfied $\theta_{k,m}=2d\frac{f_{m}}{c}\gamma_{k}$, in which *c* represents the speed of light, $\gamma_{k}=\sin{\tilde{\gamma }}_{k}\in \left[-1,1\right]$ is the physical propagation direction of the *k*-th beam (${\tilde{\gamma }}_{k}\in \left[-\frac{\pi }{2},\frac{\pi }{2}\right]$), and $d=\frac{c}{2f_{c}}$ represents the fixed antenna spacing. The frequency at *m*-th subcarrier is $f_{m}=f_{c}+\frac{B}{M}\left(m-1-\frac{M-1}{2}\right)$, where $f_{c}$ represents the center frequency and *B* represents the bandwidth. The analog precoding matrix $A\in C^{N_{t}\times K}$ generated by the classical phase-shifter network can be denoted as

(4) $$A=\left[\begin{array}{cccc} a_{1} & 0 & \cdots & 0\\ 0 & a_{2} & \cdots & 0\\ \vdots & \vdots & \ddots & \vdots \\ 0 & 0 & \cdots & a_{K} \end{array}\right],$$

where $a_{k}=\frac{1}{\sqrt{N}}{\left[1,e^{j\pi \theta_{k}},\cdots ,\;e^{j\pi \left(N-1\right)\theta_{k}}\right]}^{T}\in C^{N\times 1}$ is the analog precoding vector of the *k*-th RF chain, which is composed of frequency-independent phase shifters, and each element of $a_{k}$ should satisfy the constraint $|a_{k}\left[i,j\right]|=\frac{1}{\sqrt{N}}$ for all subcarriers. $W_{m}\in C^{N_{t}\times N_{t}}$ is the frequency-dependent phase matrix generated by the delayers, and needs to be, respectively, designed in the beam tracking and beam aggregation, which can be expressed as

(5) $$W_{m}=\left[\begin{array}{cccc} w_{1,m} & 0 & \cdots & 0\\ 0 & w_{2,m} & \cdots & 0\\ \vdots & \vdots & \ddots & \vdots \\ 0 & 0 & \cdots & w_{K,m} \end{array}\right],$$

where $w_{k,m}\in C^{N\times N}$ is the frequency-dependent phase vector of the *k*-th RF chain, and $w_{k,m}=\mathrm{diag}\left(1,e^{-j2\pi f_{m}\Delta_{m}},\cdots ,e^{-j2\pi f_{m}\left(N-1\right)\Delta_{m}}\right)$. The equivalent channel matrix of the *m*-th subcarrier $H_{m,eq}\in C^{K\times K}$ is expressed as

(6) $$H_{m}^{eq}=H_{m}W_{m}A.$$

The channel matrix singular value decomposition (SVD) is widely used in digital precoding algorithm [12]. The SVD of the equivalent channel is expressed as follows

(7) $$H_{m}^{eq}=U_{m}\Sigma_{m}V_{m}^{H},$$

where the diagonal matrix $\Sigma_{m}$ denotes the singular value of $H_{m}^{eq}$, whose main diagonal line consisting of the singular values of $H_{m}^{eq}$. $U_{m}$ and $V_{m}$ denotes the unitary matrix. Each column of the digital precoding matrix $V_{m}\in C^{K\times K}=\left[d_{1,m},d_{2,m},\cdots ,d_{K,m}\right]$ is used as digital precoding vector of each RF chain, which can make the pilot sequences of each user be orthogonal to each other, eliminate the interference among users, and satisfy the power constraint $\left|\right|Ad_{k,m}|{|}_{2}\;=\;P$, where *P* is the transmit power for each user.

## 3. Beam Tracking

**Figure 2:**
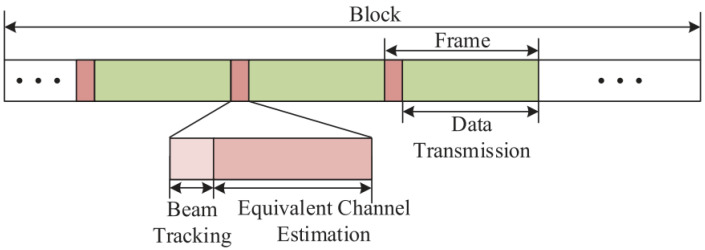
The frame structure for THz communications.

In this section, a low complexity beam tracking scheme is proposed based on the widely used frame structure of THz communication [7], as shown in Figure 2. Firstly, the time period between the successive LoS path disappearance or appearance is defined as a block, and the estimation of the full channel for each user is carried out at the beginning of each block. Then, in a block, the variation of the physical direction of the LoS path induces the change of the optimal beam in a much smaller time scale, which is defined as a frame. At the beginning of each frame, the beam tracking scheme is carried out, where new user physical directions are tracked through a beam training procedure. In the beam training procedure, the BS transmits orthogonal pilot sequences to users. In the above frame structure, the accuracy of the beam tracking scheme has a crucial impact on the achievable sum-rate performance.

The degree of beam splitting can be expanded by the delayers. Specifically, by jointly controlling time delay and analog precoding parameters, the beam of each RF chain is split into *M* sub-beams corresponding to *M* subcarriers, respectively, which can cover the whole tracking range aiming at detecting different user’s physical directions, so the beam tracking problem can be transformed into the low-complexity beam selection problem. The equivalent analog precoding vector of the *m*-th subcarrier on the *k*-th RF chain is

(8) $$\begin{array}{c} \;a_{k,eq}=w_{k,m}a_{k}\\ \;=\mathrm{diag}\left(1,e^{-j2\pi f_{m}\Delta_{m}},\cdots ,e^{-j2\pi f_{m}\left(N-1\right)\Delta_{m}}\right)\times \frac{1}{\sqrt{N}}{\left[1,e^{j\pi \theta_{k}},\cdots ,e^{j\pi \left(N-1\right)\theta_{k}}\right]}^{T}\\ =\frac{1}{\sqrt{N}}{\left[1,e^{j\pi (\theta_{k}-2f_{m}\Delta_{m})},\cdots ,e^{j\pi \left(N-1\right)\left(\theta_{k}-2f_{m}\Delta_{m}\right)}\right]}^{T}. \end{array}$$

Then, the equivalent beam space direction of the *m*-th subcarrier on the *k*-th RF chain can be obtained as

(9) $$\theta_{k,m}^{eq}=\theta_{k}-2f_{m}\Delta_{m},$$

where $\theta_{k,m}^{eq}=\frac{2d}{c}f_{m}\gamma_{k,m}^{eq}$, in which the equivalent physical direction is defined as $\gamma_{k,m}^{eq}=1-\frac{2}{M-1}\left(m-1\right)\in \left[-1,1\right]$, and $\theta_{k}$ can be determined as $\frac{2d}{c}f_{M}\gamma_{M}$, where $\gamma_{M}=1$. In view of $f_{M}\gamma_{M}=f_{m}\gamma_{m}$[7,8], then $\theta_{k}=\frac{2d}{c}f_{m}\left(\frac{f_{M}}{f_{m}}\gamma_{M}\right)$. By redefining $f_{m}=f_{M}-\frac{B}{M}\left(M-m\right)$, Equation (9) can be further expressed as

(10) $$\begin{array}{c} \frac{2d}{c}f_{m}\gamma_{k,m}^{eq}=\frac{2d}{c}f_{m}\left(\frac{f_{M}}{f_{m}}\gamma_{M}\right)-2f_{m}\Delta_{m}\;\\ \Rightarrow \Delta_{m}=\frac{d}{c}\left(\frac{f_{M}}{f_{m}}\gamma_{M}-\gamma_{k,m}^{eq}\right)\;\\ \;\;=\frac{1}{2f_{c}}\left(\frac{1}{1-\frac{B}{f_{M}M}\left(M-m\right)}+\frac{2}{M-1}\left(m-1\right)-1\right).\; \end{array}$$

Considering $\frac{B}{f_{M}M}\left(M-m\right)<1$, the value of $\Delta_{m}$ is positive. In addition, $\Delta_{m}$ is composed of $B/f_{M}$, so it can adapt to beam splitting under different-bandwidth frequency ratios. Through the design of time delay and analog precoding, the beam of each RF chain is split into *M* sub-beams corresponding to *M* subcarriers, respectively, providing the feasibility for transforming the beam tracking problem into a low complexity beam selection problem. Then, by using the beam selection method based on maximum magnitude (MM) [13], the physical direction $\gamma_{k}^{{}^{\prime }}$ and the sub-beam number (i.e., the subcarrier number *m*) of the *k*-th user can be determined. This provides a necessary premise for the subsequent beam aggregation.

The advantages of the proposed HP in beam tracking are as follows: (1) One RF chain generates a naturally split beam with *M* different directions corresponding to *M* subcarriers. The naturally split beam with limited physical range can be expanded into a beam with *M* equal interval sub-beams, that can cover the whole tracking range (i.e., $\gamma_{k,m}^{eq}\in \left[-1,1\right]$), through the design of time delay and analog precoding. This design can improve the accuracy of beam tracking. (2) Multiple RF chains are required for multi-user beam tracking. Based on the orthogonality of digital precoding, beams with equal interval and global physical direction for multiple users can be generated at the same time, and simultaneous beam tracking for multiple users can be performed. This design can greatly improve the efficiency of beam tracking.

## 4. Beam Aggregation

Based on the processing of beam tracking above, the physical direction of each user $\gamma_{k}^{{}^{\prime }}$ is already known. Because the system’s achievable sum-rate performance is directly related to the array gain, the achievable rate maximization problem can be transformed into the array gain maximization problem. Through the design of time delay and analog precoding, the degree of beam splitting can be reduced and the *M* naturally split sub-beams on each RF link can be aggregated in the corresponding user physical direction. This can maximize the array gain at last. The normalized array gain of the *k*-th sub-array of the *m*-th subcarrier at the base station is

(11) $$\begin{array}{c} f_{t}^{H}\left(\theta_{k,m}\right)w_{k,m}a_{k}\;\\ =\frac{1}{\sqrt{N}}\left[1,e^{-j\pi \theta_{k,m}},e^{-j\pi 2\theta_{k,m}},\cdots ,e^{-j\pi \left(N-1\right)\theta_{k,m}}\right]\;\\ \;\;\times \mathrm{diag}\left(1,e^{-j2\pi f_{m}\Delta_{m}},\cdots ,e^{-j2\pi f_{m}\left(N-1\right)\Delta_{m}}\right)\times \frac{1}{\sqrt{N}}{\left[1,e^{j\pi \theta_{k}},\cdots ,e^{j\pi \left(N-1\right)\theta_{k}}\right]}^{T}\;\\ =\frac{1}{N}\left(1+e^{j\pi (\theta_{k}-\theta_{k,m}-2f_{m}\Delta_{m})}+\cdots ,+e^{j\pi \left(N-1\right)\left(\theta_{k}-\theta_{k,m}-2f_{m}\Delta_{m}\right)}\right). \end{array}$$

To maximize Equation (11), we make $\theta_{k}-\theta_{k,m}-2f_{m}\Delta_{m}=0$. By recalling the definition of $\theta_{k}$ and $\theta_{k,m}$ determined in beam tracking, when $\gamma_{k}^{{}^{\prime }}\in \left[0,1\right]$, we can obtain

(12) $$\begin{array}{c} \Delta_{m}=\frac{1}{2f_{m}}\left(\theta_{k}-\theta_{k,m}\right)\;\\ =\frac{1}{2f_{m}}\left(2df_{m}\left(\frac{f_{M}}{f_{m}c}\gamma_{k}^{{}^{\prime }}\right)-2d\frac{f_{m}}{c}\gamma_{k}^{{}^{\prime }}\right)\;\\ =\frac{1}{2f_{c}}\gamma_{k}^{{}^{\prime }}\left(\frac{1}{1-\frac{B}{f_{M}M}\left(M-m\right)}-1\right). \end{array}$$

When $\gamma_{k}^{{}^{\prime }}\in \left[-1,0\right)$, $\theta_{k}$ can be determined as $\frac{2d}{c}f_{1}\gamma_{k}^{{}^{\prime }}$, and we redefine $f_{m}=f_{1}+\frac{B}{M}\left(m-1\right)$, it can be obtained

(13) $$\begin{array}{c} \Delta_{m}=\frac{1}{2f_{m}}\left(\theta_{k}-\theta_{k,m}\right)\;\\ =\frac{1}{2f_{m}}\left(2df_{m}\left(\frac{f_{1}}{f_{m}c}\gamma_{k}^{{}^{\prime }}\right)-2d\frac{f_{m}}{c}\gamma_{k}^{{}^{\prime }}\right)\;\\ =\frac{1}{2f_{c}}\gamma_{k}^{{}^{\prime }}\left(\frac{1}{1+\frac{B}{f_{1}M}\left(m-1\right)}-1\right).\; \end{array}$$

After the analog precoding is determined, the value of $\Delta_{m}$ in both Equations (12) and (13) are non-negative. In addition, the expression of $\Delta_{m}$ contains $B/f_{M}$ and $B/f_{1}$, respectively, so it can adapt to different bandwidth-frequency ratios in beam aggregation.

## 5. Simulation Results

In order to make a fair comparison with the schemes proposed in [7,8], the system parameters are set as $f_{c}=100\;\mathrm{GHz}$, $N_{t}=1024$, M=128, K=4. Equation (14) is used to calculate the average achievable sum-rate of one subcarrier in each scheme, where the signal-to-noise ratio is $\mathrm{SNR}=P/\sigma^{2}$.

(14) $$\begin{array}{c} R=\frac{1}{M}\sum_{m=1}^{M}\mathrm{lo}g_{2}\left(\right|I_{K}+\frac{P}{K\sigma^{2}}H_{m}W_{m}AD_{m}D_{m}^{H}A^{H}W_{m}^{H}H_{m}^{H}\left|\right). \end{array}$$

**Figure 3:**
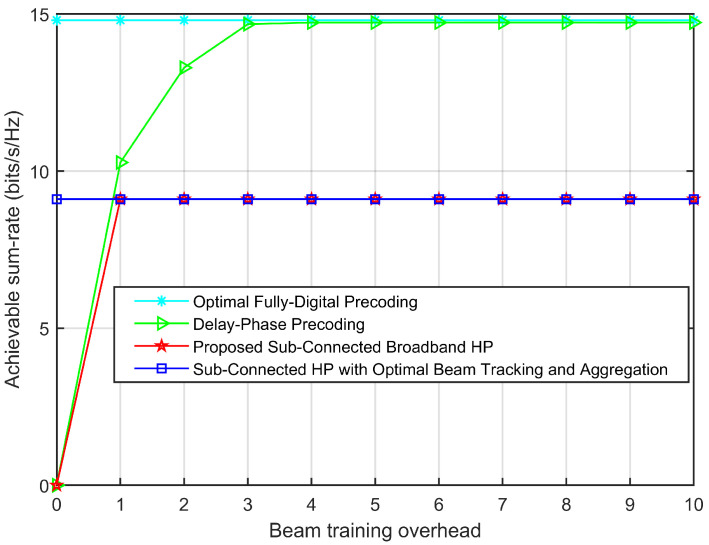
Effect of beam tracking on achievable sum-rate (SNR=10dB).

The effect of beam tracking on achievable sum-rate (SNR=10dB) is shown in Figure 3. Where sub-connected HP [10] and optimal fully-digital precoding [14] are respectively used as the best performance benchmarks of the classical sub-connected HP structure and the classical fully-connected HP structure. The lateral axis shows the beam training overhead (i.e., the number of time slots required for beam tracking). In order to highlight the influence of beam tracking on achievable sum-rate, the effect of beam aggregation is ignored, so B=1GHz is set. It can be seen from Figure 3 that, the proposed HP is based on a sub-connected structure, while the delay-phase precoding [7] is based on a fully-connected structure, so the performance of achievable sum-rate is weaker in the former than in the latter. However, the proposed HP has a faster and more accurate performance than that of the delay-phase precoding (The proposed beam tracking scheme only needs one time slot to achieve simultaneous tracking of each user’s physical direction). This is because the proposed HP transforms the beam tracking problem into a low-complexity beam selection problem. Furthermore, different from the beam tracking scheme of delay-phase precoding, the proposed beam tracking scheme does not require a priori information of the user moving variation range.

**Figure 4:**
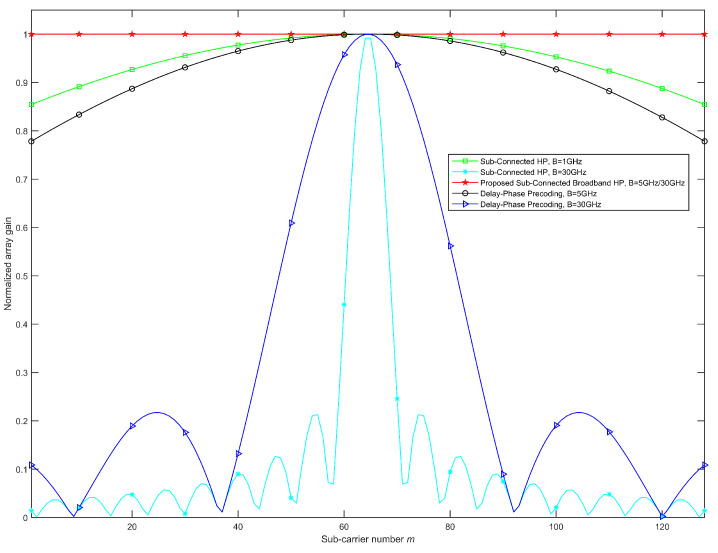
Normalized array gain of each subcarrier at different bandwidth-frequency ratios in beam aggregation.

The normalized array gain of each subcarrier at different bandwidth-frequency ratios ($B/f_{c}$) in beam aggregation is shown in Figure 4. It can be seen from Figure 4 that, when $f_{c}=100\;\mathrm{GHz}$, as the bandwidth increases, the beam splitting question becomes more and more serious. Under this circumstance, the classical hybrid precoding and the delay-phase precoding can only achieve 100% array gain on a very small number of subcarriers, while most subcarriers will suffer severe array gain loss. However, the proposed HP can achieve 100% array gain at all subcarriers with different bandwidth-frequency ratios, so it can be considered as an optimal solution to deal with the beam splitting problem.

**Figure 5:**
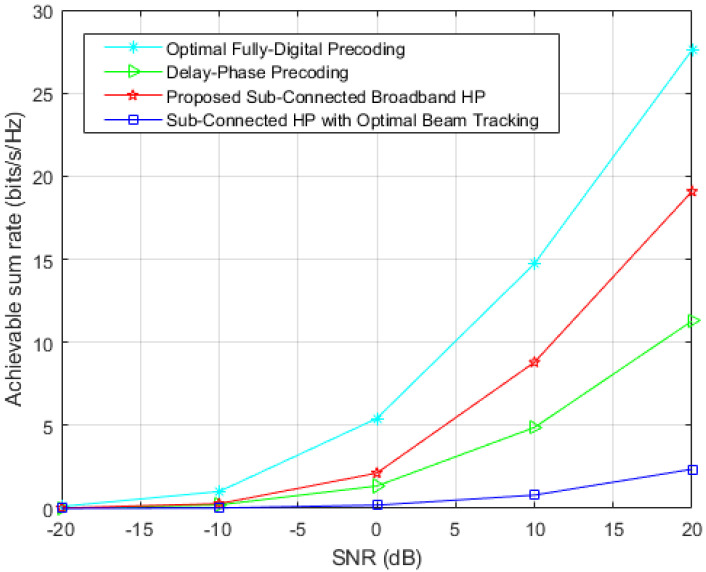
Comparison of achievable sum-rate of different precoding under high bandwidth-frequency ratio (B=30GHz).

The achievable sum-rate comparison among the proposed HP scheme and other existing methods under high bandwidth-frequency ratio (B=30GHz) is shown in Figure 5, and the implementation process includes beam tracking and beam aggregation. It can be seen from Figure 5 that, compared with the proposed HP, both the classical sub-connected HP with optimal beam tracking and the delay-phase precoding [8] cannot effectively deal with the degradation in achievable sum-rate performance caused by beam splitting at high bandwidth-frequency ratios, even if the delay-phase precoding can achieve near-optimal achievable sum-rate performance at the low bandwidth-frequency ratios (in Figure 3, *B* = 1 GHz can be seen as a case where beam splitting does not exist or a perfect solution is adopted.). The main reason for this phenomenon is that the large bandwidth is not considered in the design of time delay, so the effect is very limited under the large bandwidth-frequency ratio. However, the proposed HP can perfectly compensate for the loss of achievable sum-rate caused by beam splitting questions in beam aggregation, and obtain the best achievable sum-rate performance of hybrid precoding with sub-connected structure.

Energy efficiency (EE) is affected by these factors: achievable sum-rate *R*, total transmit power $P_{t}$, baseband power $P_{\mathrm{BB}}$, RF chain power consumption $P_{\mathrm{RF}}$, phase shifter power consumption $P_{\mathrm{PS}}$, and delayer power consumption $P_{\mathrm{TD}}$, etc. The energy efficiency of the proposed HP is expressed as

(15) $$E=\frac{R}{P_{t}+P_{\mathrm{BB}}+KP_{\mathrm{RF}}+N_{t}P_{\mathrm{TD}}+N_{t}P_{\mathrm{PS}}}.$$

The energy efficiency of the delay-phase precoding $E_{D}$ is expressed as

(16) $$E_{D}=\frac{R_{\mathrm{DPP}}}{P_{t}+P_{\mathrm{BB}}+KP_{\mathrm{RF}}+KN_{\mathrm{TD}}P_{\mathrm{TD}}+KN_{t}P_{\mathrm{PS}}},$$

where $N_{\mathrm{TD}}$ is the number of the delayers used in one RF chain in delay-phase precoding.

**Figure 6:**
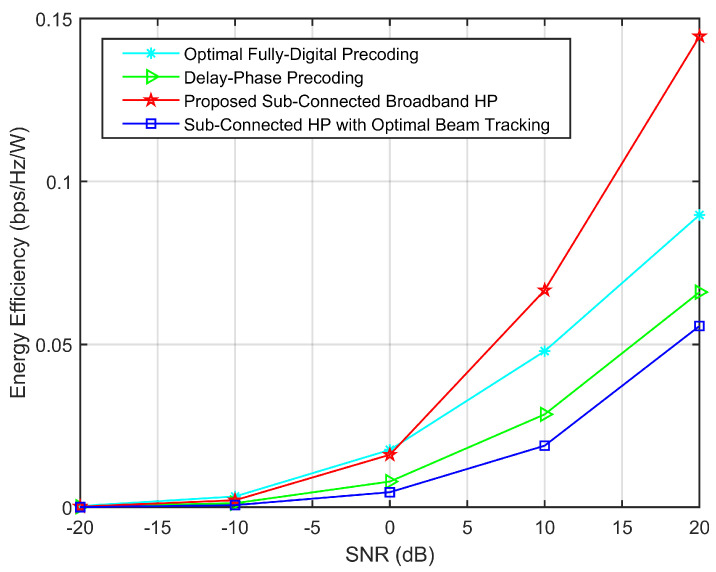
Comparison of energy efficiency of different precoding under high bandwidth-frequency ratio (*B* = 30 GHz).

The comparison of energy efficiency of different precoding under a high bandwidth-frequency ratio (*B* = 30 GHz) is shown in Figure 6. We adopt $P_{t}=32\;\mathrm{mW}$, $P_{\mathrm{RF}}=300\;\mathrm{mW}$, $P_{\mathrm{BB}}=200\;\mathrm{mW}$, $P_{\mathrm{PS}}=40\;\mathrm{mW}$ and $P_{\mathrm{TD}}=87.5\;\mathrm{mW}$[9]. It can be seen from Figure 6 that, the proposed HP enjoys higher energy efficiency than other hybrid precodings. Here, the number of the delayers in the delay-phase precoding is $KN_{\mathrm{TD}}$, while in the proposed HP is $N_{t}$. Although the energy consumption of the delayed-phase precoding is much lower than that of the proposed HP and fully-digital precoding scheme, the achievable sum-rate performance of which is seriously degraded. Since the beam splitting question under a high bandwidth-frequency ratio cannot be effectively solved, resulting in a decline of energy efficiency, the energy efficiency of the delayed-phase precoding is even lower than that of fully-digital precoding when $N_{\mathrm{TD}}=16$ (i.e., the best experience value in [7,8]). The energy efficiency of the proposed HP is higher when SNR becomes larger, while it is equal to fully-digital precoding in low SNR. Since the beam tracking will be more accurate with the SNR increases, which can fully eliminate the beam splitting by beam aggregation.

## 6. Conclusions

A broadband HP scheme based on adaptive beam splitting is proposed. The frequency-dependent phase shift is introduced through the delayers. After time delay and analog precoding are designed, the broadband beam with flexible and controllable coverage in the angle domain can be generated. In beam tracking, simultaneous and global tracking of multiple users can be realized, thus greatly improving beam tracking efficiency. In beam aggregation, accurate aggregation of naturally split sub-beams can be realized, thus greatly improving the achievable sum-rate performance. The simulation results verify the effectiveness of the proposed HP in beam tracking and beam aggregation, respectively. 

## Data Availability

Not applicable.
